# Protective effect of a JNK inhibitor against retinal ganglion cell loss induced by acute moderate ocular hypertension

**Published:** 2011-04-06

**Authors:** Hui Sun, Ying Wang, Iok-Hou Pang, Jiaquan Shen, Xia Tang, Ying Li, Chuanyong Liu, Bing Li

**Affiliations:** 1 Department of Physiology, Shandong University School of Medicine, Shandong, P.R. China; 2 Department of Physiology, Binzhou Medical College, Binzhou, Shandong, P.R. China; 3 Department of Ophthalmology, Shanghai first People’s Hospital, Shanghai Jiaotong University School of Medicine, Shanghai, China; 4 Glaucoma Research, Alcon Research, Ltd., Fort Worth, TX; 5 Department of Ophthalmology, Shandong University School of Medicine, Shandong, P.R. China; 6 Key Lab of Medical Neurobiology of Shandong Province, Jinan, Shandong, P.R. China

## Abstract

**Purpose:**

To correlate retinal ganglion cell (RGC) loss and optic nerve (ON) damage with the duration of acute glaucoma attacks in a rat experimental model and to determine whether the c-Jun N-terminal kinase (JNK) inhibitor SP600125 protects against such attacks.

**Methods:**

To model an acute glaucoma attack, rat intraocular pressure (IOP) was elevated by a controllable compression method using pulleys and specific weights. Intraocular pressure was measured with a TonoLab® rebound tonometer. Time-dependent ocular hypertension-induced damage was evaluated by ON morphology, retina morphology (both retina layer thickness in cross-sections and RGC counts in Dextran tetramethylrhodamine crystals [DTMR] labeled flatmounts), and scotopic flash electroretinography (ERG). A c-Jun N-terminal kinase (JNK) inhibitor, SP600125 (0, 1.5, 5, or 15 mg/kg), was administered by intraperitoneal injection immediately before and after induction of ocular hypertension, then once daily for seven days. Retinal cross-sections were measured to determine the thickness of various retinal layers and the cell density in the ganglion cell layer (GCL). Retinal flatmounts immunolabeled with anti-rat Brn-3a primary antibody were used to quantify RGC numbers.

**Results:**

Elevated rat IOP induced by corneal limbus compression correlated with the different weights. Elevation to 45 mmHg for up to 7 h did not significantly affect the thicknesses of the outer nuclear layer, outer plexiform layer, or inner nuclear layer. Amplitudes of A- and B-waves were not affected. However, elevation to 45 mmHg for up to 7 h decreased the inner retinal thickness and caused ON damage. Most importantly, IOP elevation induced a time-dependent RGC loss. Cell density in the GCL decreased to 70%, 62%, and 49% of that of the control after 5 h, 6 h, and 7 h, respectively, of pressure increases. In retinal flatmount studies, labeled RGCs were reduced 56±4% (mean±SEM) versus the control (p<0.001) after 7 h of ocular hypertension. SP600125 dose-dependently protected against ocular hypertension-induced RGC loss. The difference in RGC density between the vehicle and SP600125-treated (15 mg/kg) groups was statistically significant (p<0.001).

**Conclusions:**

The correlation of inner retinal morphological changes with the duration of the application of 45 mmHg IOP was demonstrated. Treatment with SP600125 significantly protected RGC survival against this insult. Inhibitors of JNK may be an interesting pharmacological class for treating glaucoma.

## Introduction

Glaucoma is one of the most prevalent causes of irreversible blindness in the world. It is estimated that in 2010 there were 60.5 million glaucoma patients worldwide, with 44.7 million affected by primary open angle glaucoma (POAG) and 15.7 million affected by primary angle-closure glaucoma (PACG). In the next 10 years, the total number of PACG patients will increase to 21 million; of those, 5.3 million will be bilaterally blind [1]. A major risk factor for glaucomatous damage is elevated intraocular pressure (IOP). Retinal ganglion cells (RGCs) are the retinal components most sensitive to IOP elevation; RGC damage is responsible for the loss of vision in glaucoma. As a medical emergency, the IOP of eyes with acute angle-closure glaucoma can be as high as 40–80 mmHg, which is believed to result in permanent vision loss if not treated within hours of the attack [2,3]. To induce selective damage in the inner retinal layers in animal models, many studies have demonstrated that an IOP elevation to 30–50 mmHg is necessary. This causes selective damage in the inner retinal layers, such as a reduced scotopic threshold response (STR), photopic negative response (PhNR), and amplitude of the pattern electroretinogram (PERG) [4-10].

In recent years, many animal glaucoma models have been established [11]. However, almost all these models were designed to study POAG; they either induce a low level but prolonged IOP elevation, or generate RGC damage via insults unrelated to pressure. These models typically do not address the biologic changes and potential therapeutic approaches related to acute PACG attacks. So far, the induced changes of the inner retinal layer by transient acute moderate elevation of IOP are reversible [4,12-14], which is quite different from the irreversible functional, RGC, and inner retinal changes seen in acute glaucoma attacks. We believe that, in addition to moderately elevated IOP, the duration of the elevation is another key factor in inducing damage of RGCs in an animal study. To do this, we induced a controllable, moderate elevation in IOP using a suture-pulley model for several hours and monitored changes in the retina and optic nerve (ON), which provides important insight into the pathology of an acute PACG attack. As previously reported [13], the suture-pulley method uses sutures that loop around and compress the external corneal limbal region to produce rat ocular hypertension, the magnitude of which depends on the weights attached to the ends of the suture. In the present study, we characterized the relationship between the applied weights and IOP elevation and the effects of ocular hypertension on the functional and morphological changes in the retina, thereby damaging retinal components in a more selective and controllable fashion.

We further evaluated the usefulness of this method in assessing a potential neuroprotective agent, an inhibitor of c-Jun N-terminal kinase (JNK). Being a member of the mitogen-activated protein kinase family, JNK is involved in the signal transduction of a variety of cellular pathways, including apoptosis, inflammation, and carcinogenesis [15-17]. Phosphorylation of JNK and activation of its signaling cascade have been demonstrated during RGC apoptosis in experimental open angle glaucoma (OAG) [18]. Thus, the blockade of this pathway by specific inhibitors may prevent or slow the progression of RGC loss in the current PACG attack model. SP600125 is a specific, commonly used JNK inhibitor. It has been demonstrated to reverse neuronal cell death in rat hippocampal Cornu Ammonis 1 (CA1) caused by transient brain ischemia/reperfusion [19,20]. In RGC apoptosis induced by N-Methyl-D-aspartic acid or N-Methyl-D-aspartate (NMDA), the expression of JNK increased and SP600125 reversed the apoptotic process [21]. In a preliminary report, we demonstrated that the p-JNK pathway was activated by applying IOP of 45 mmHg over 6 h and was blocked by SP600125 in the ganglion cell layer (GCL) [22]. Hence, in the current study, we investigated whether SP600125 would prevent RGC loss induced by ocular hypertension.

## Methods

Procedures used in this investigation conformed to the Association for Research in Vision and Ophthalmology (ARVO) resolution on the Use of Animals in Ophthalmic and Vision Research and were approved by the Animal Care and Use Committee at Shandong University School of Medicine in China. Male Wistar rats weighing 200–250 g were purchased from the Animal Center at Shandong University. They were housed in rooms in which the temperature, humidity, and lighting (12 h:12 h light-dark cycle) were controlled and water and food were available ad libitum.

### Elevation of IOP

Acute unilateral elevated IOP was induced by the suture-pulley corneal limbal compression method described previously [13]. Briefly, rats were anesthetized with chloral hydrate (400 mg/kg, intraperitoneal), with additional doses (60 mg/kg) given as needed. A suture thread (1/0 to 4/0 silk) of approximately 70 cm was connected to the indicated weights at both ends. The thread was then looped around the circumference of the eyeball approximately 2 mm behind the limbus. Circumferential compression of the globe symmetric to the optical axis was produced by passing both ends of the suture thread through a series of pulleys. The contralateral untreated eye served as a naïve control.

To confirm continuous ocular hypertension in the eye, IOP was measured using a TonoLab® rebound tonometer (Colonial Medical Supply, Franconia, NH) at 5 min before IOP elevation, then every 15 min for the first 120 min of IOP elevation, and every 60 min for the remaining period of elevation. The elevated IOP was maintained for the indicated duration and up to 7 h. Throughout the procedure, the mean arterial blood pressure was monitored and reported by a Powerlab/8SP data acquisition system (ML785; ADInstrument, Bella Vista, Australia).

### Evaluation of optic nerve damage

Four weeks after ocular hypertension, the animals were euthanized. The optic nerve (ON) of each eye was isolated and fixed immediately in 2% paraformaldehyde and 2.5% glutaraldehyde in a 0.1 M cacodylate buffer (pH 7.2) overnight, placed in 1% OsO_4_ and in 0.25% uranyl acetate for 2 h each, dehydrated with a series of acetones, and then embedded in epoxy resin (Epon Araldite; Mirivac, Halifax, NS, Canada). Next, 1 µm sections were cut, placed on glass slides, and stained with 1% toluidine blue (Sigma-Aldrich, Oakville, ON, Canada). Stained sections were photographed at 10× magnification using a digital camera (Eclipse 80i; Nikon, Tokyo, Japan) and printed so the whole nerve was visible in the field of view. The severity of ON damage in each section was independently graded by three masked investigators using an Optic Nerve Damage Score (ONDS) [23], as follows: Grade 1=normal; Grade 2=up to 20% dead and darkly stained axons with initial gliosis; Grade 3=up to 50% dead axons with mild gliosis; Grade 4=up to 80% dead axons with prominent gliosis; and Grade 5=almost 100% dead axons with severe gliosis. The mean ONDS of each ON determined by the three investigators was reported and evaluated using statistical analysis.

### Histopathology of retinal cross-sections

Eyeballs of euthanized rat were fixed in 4% paraformaldehyde overnight and embedded in paraffin. Next, 4 µm thick sections were cut across the optic papilla and stained with hematoxylin and eosin. For quantitative analyses, sections perpendicular to the retinal surface were examined under a stereomicroscope (Eclipse 80i; Nikon). Thicknesses of five retinal layers were measured in a masked fashion at three adjacent areas within 0.5 mm of the ON in the inferior peripapillary region and the mean values were reported. The five retinal layers are: 1) overall retinal thickness from the outer limiting membrane to the inner limiting membrane, 2) the outer nuclear layer (ONL), 3) the outer plexiform layer (OPL), 4) the inner nuclear layer (INL), and 5) inner retinal thickness from the inner plexiform layer to the limiting membrane (IPL-ILM). Measurements were performed in the same topographic region of the retina to minimize regional anatomic variations.

Cell counts of the GCLs were performed manually across a length of 300 µm in the same topographic region of the retina.

### Quantification of DTMR-labeled RGCs in Retina Flatmounts

Twenty-four hours before euthanasia, rats were anesthetized with a cocktail of ketamine (75 mg/kg) and xylazine (5 mg/kg) and their ONs were completely transected at about 2 mm behind the globe, without injuring the ophthalmic artery. Dextran tetramethylrhodamine crystals (DTMR; 3000 molecular weight; Molecular Probes, Eugene, OR) were applied at the cut end of the ON stump. Twenty-four hours later, eyes were enucleated and fixed in a 4% paraformaldehyde solution at 4 °C for 120 min. The retinas were dissected from the eye cups and prepared as flatmounts, with four radially-oriented cuts in each retina. These were then whole-mounted on glass slides. The slides were kept in the dark and were air dried overnight. The tissue was protected by a cover glass with mounting medium for fluorescence (Vector, Burlingame, CA). The DTMR-labeled RGCs were viewed using a fluorescence microscope (Leica) with rhodamine filters with maximal absorption at 560 nm. Digital photos of each retina were taken in a low-light room using imaging processing software (MegnaFire version 2.1). Images of one central and one peripheral field (40× magnification; area of each field=0.092 mm^2^) were captured from each of the four retinal quadrants and were printed on a color printer. The labeled RGC numbers of each color image print were manually counted by an observer masked to the protocol. The cell counts of each image were then converted into cells per square millimeter. The cell density of each eye was calculated by averaging the cell numbers counted from eight image areas of each retina. Next, RGC loss in the experimental eye was calculated as percentage of cell loss compared to the control eye.

### Brn-3a immunolabeling of RGCs in retina flatmounts

The methods for Brn-3a immunolabeling of RGCs have been previously described [24]. Briefly, enucleated eyeballs were fixed in a 4% paraformaldehyde solution at 4 °C for 120 min. A cut was made through the corneoscleral limbus. The retinas were treated sequentially with 10%, 20%, for 60min each, and then overnight with 30% sucrose and were then frozen and thawed three times, washed with PBS, incubated in 10% methanol-3% H_2_O_2_-PBS for 30 min, and blocked with 2% BSA in PBS for 2 h. Treated retinas were then incubated overnight with monoclonal mouse anti-rat Brn-3a primary antibody (1:50; Chemicon, Temecula, CA) and were then incubated with horse anti-mouse IgG H^+^L secondary antibody (1:200; Vecter, Burlingame, CA) for 2 h after being washed in PBS. Retinas were incubated in Extravidin solution at room temperature for 2 h in the dark. Following PBS washing (10 min/each), each retina was incubated using a PharMingen™ DAB substrate Kit (BD Biosciences, San Jose, CA) until the desired color intensity developed. Stained retinas were flatmounted, microscopic images were captured, and cell counts were analyzed, similar to the DTMR-labeled retina flatmounts.

### Electroretinography

Scotopic ERG was used to assess potential damage to the outer retinal layer by the elevated IOP [13]. Briefly, animals were dark-adapted overnight and anesthetized. The pupils were dilated with Mydfrin™ (Alcon, Fort Worth, TX) and corneas were anaesthetized with Alcain (Alcon). White light flashes were produced by a photostimulator (Model PS22; Grass Instruments, West Warwick, RI) placed 25 cm in front of the rat’s eye. The responses were recorded and analyzed by data wave electroretinogram collection software. Baselines of A- and B-wave amplitudes were collected before IOP was elevated. They were used as a comparison against the respective ERG values collected at the indicated time point after IOP elevation.

### Administration of test articles

SP600125 (Sigma) was dissolved in DMSO and diluted with 0.01 M PBS (154 mM NaCl,1.9 mM NaH_2_PO_4_,8.1 mM Na_2_HPO_4_, pH 7.4) to a final concentration of 1, 3.3, and 10 mg/ml (containing 1% DMSO). SP600125 (1.5, 5, and 15 mg/kg) or the same volume of vehicle was administrated intraperitoneally for a total of seven doses, at 5 min before and immediately after IOP elevation, and then once daily on Days 2–7 after IOP elevation.

### Statistical analysis

Data are presented as mean±SEM and were analyzed with SigmaStat 3.5 software (SPSS Inc., Chicago, IL). A one-way ANOVA, followed by a Dunnett’s or Bonferroni’s test was used to compare results among three study groups. A p<0.05 was considered statistically significant.

## Results

### Intraocular pressure elevation

**Figure 1:**
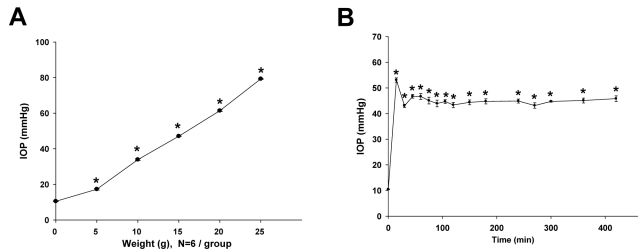
Intraocular pressure (IOP) elevation using the suture-pulley technique. **A**: Relationship of rat IOP and weights attached to the ends of the suture. The IOP was measured for 60 min after the indicated weights were attached. Each symbol represents mean±SEM (n=6). *: p<0.001 versus baseline using a one-way ANOVA and a Dunnett’s test. **B**: Effect of using 15 g weight on rat IOP. The weight was applied starting at Time 0 and was maintained for 7 h for anesthetized rats. Each symbol represents mean±SEM (n=31 from Time 0 to 360 min, n=17 from 360 min to 420 min). *: p<0.001 versus baseline using a one-way ANOVA and a Dunnett’s test.

As previously reported, the suture-pulley method produces rat ocular hypertension, the magnitude of which depends on the weights attached to the ends of the suture [13]. Therefore, when the standard weight increases, IOP increases correspondingly. In this study, the IOP of anesthetized rats before application of the weight was 10.5±0.2 mmHg. At 60 min after a 5 g weight was applied, the IOP was elevated to 17.3±0.3 mmHg. Similarly, the IOP was increased to 33.9±0.5 mmHg by 10 g, 47.0±0.1 mmHg by 15 g, 61.4±0.5 mmHg by 20 g, and 79.3±0.3 mmHg by 25 g (Figure 1A). Based on these results and because of the moderate IOP elevation it produced, 15 g of weight was chosen for the rest of the study.

When 15 g of weight was applied, the rat IOP peaked transiently to 53.0±1.3 mmHg and stabilized at 45.0±0.1 mmHg (range from 43 to 47 mmHg) until the weight was removed at 7 h (Figure 1B). During the experiment period, no retinal blanching was observed by ophthalmoscopy. However, between 1 and 2 h during the procedure, the lens became partially cloudy, which lasted for approximately an hour before clearing. No other anomaly was noted. The IOP of the contralateral eye was maintained at the baseline level. The mean arterial blood pressure did not significantly change during the 7 h study period (data not shown).

### Optic Nerve Damage Induced by IOP Elevation

**Figure 2:**
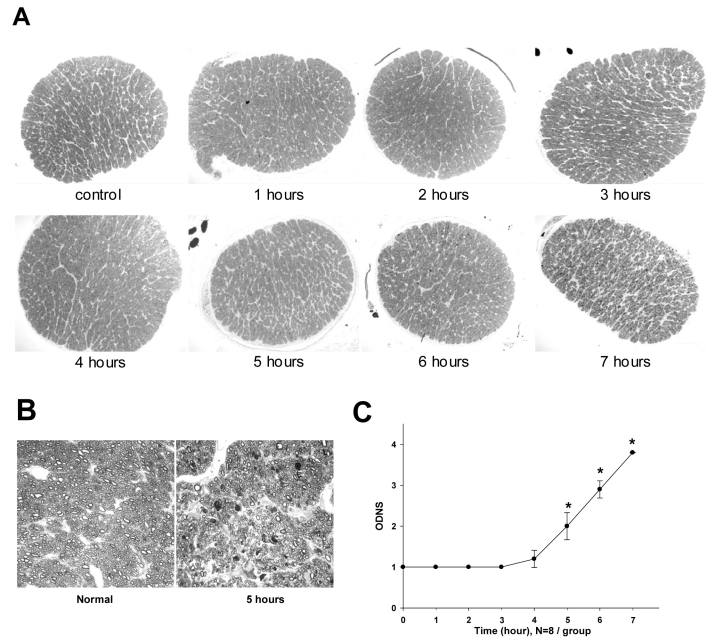
Time-dependent histological changes of rat optic nerves (ONs) induced by ocular hypertension (45 mmHg). Evaluation was performed four weeks after the injury. **A**: Representative images of cross-sections of ONs from rats treated with 0 (control), 1, 2, 3, 4, 5, 6, or 7 h of elevated intraocular pressure (IOP; 45 mmHg). In the control, which was subjected to 1–4 h of pressure, there was no identifiable axonal swelling or damage or gliosis in the ON. When the duration of ocular hypertension was 5 h or longer, there was apparent axonal damage (darkly stained axons) and swelling, and gliosis (lightly stained) became obvious. **B**: Higher magnification images of an ON from a control rat and one that had elevated IOP for 5 h, showing axonal damage. **C**: Semi-quantitative analysis of pressure-induced ON damage using the Optic Nerve Damage Score (ONDS). Each symbol represents mean±SEM (n=8). *p<0.001 versus the control group using a one-way ANOVA and a Dunnett’s test.

To evaluate the ON damage in rats subjected to 1–7 h of IOP elevation (45 mmHg) 28 days after the insult, the morphology of the corresponding ON was assessed and an ONDS was assigned. Representative images from all groups are shown in Figure 2A, as are two higher magnification images of an ON from a control rat and one that had elevated IOP for 5 h. These images show a duration-dependent injury of the ON. No significant morphological changes were found in the ON of the 1 h, 2 h, 3 h, and 4 h groups. However, mild damage (ONDS=2±0.33, p<0.05) in the 5 h group, an obvious injury (ONDS=2.9±0.21, p<0.01) in the 6 h group, and very significant damage (ONDS=3.8±0.07, p<0.001) in the 7 h group was observed (Figure 2C).

### Changes in Retinal Layers Induced by IOP Elevation

**Figure 3:**
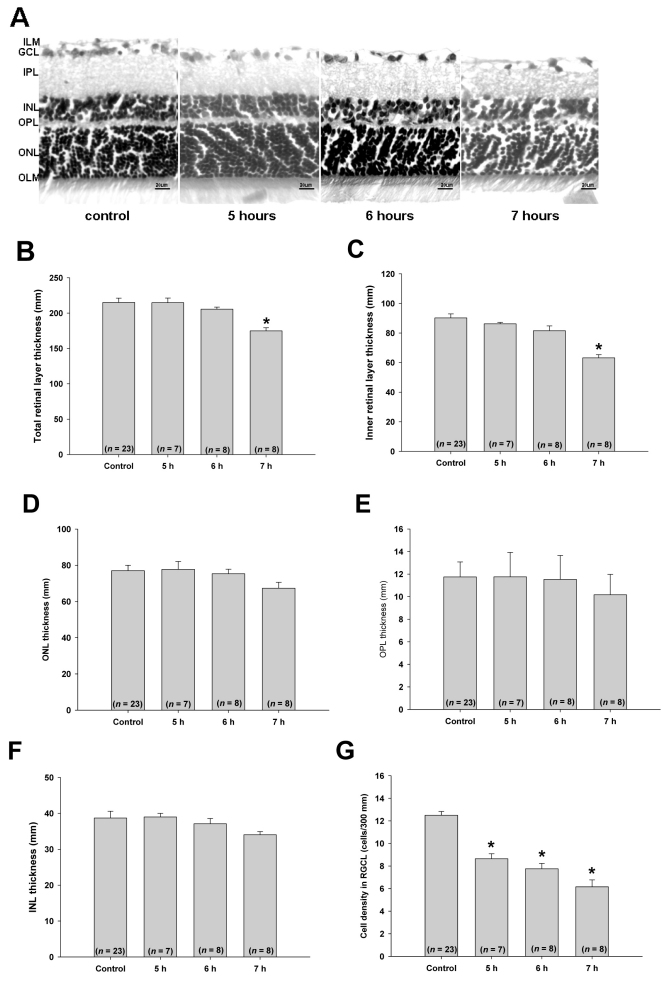
Time-dependent morphological changes of the rat retina induced by ocular hypertension (IOP=45 mmHg). **A**: Representative images of retina cross-sections from animals with 5, 6, or 7 h of induced ocular hypertension. The sections were stained with hematoxylin and eosin and the thicknesses of various retinal layers and cell densities in the ganglion cell layer (GCL) were measured. Untreated eyes served as controls. Thicknesses of the overall retina [OLM-ILM] (**B**), inner retinal layer [IPL-ILM] (**C**), ONL (**D**), OPL (**E**), INL (**F**), and cell density in the GCL (**G**) were quantified and are shown. Bars represent mean±SEM *: p<0.001 versus control using a one-way ANOVA and a Dunnett’s test. GCL: ganglion cell layer; ILM: inner limiting membrane; INL: inner nuclear layer; IPL: inner plexiform layer; OLM: outer limiting membrane; ONL: outer nuclear layer; OPL: outer plexiform layer.

At Day 28, retinas that experienced 5 h, 6 h, or 7 h of ocular hypertension were examined for morphological changes. Representative images of treated retinas are shown in Figure 3A. These images show a duration-dependent reduction in GCL cell density and thinning of the inner retinal layer after 7 h of IOP elevation. Quantification of these changes demonstrated that overall retinal thickness (OLM to ILM) did not change significantly, except in the 7 h IOP elevation group. Thickness in the control group was 215.1±1.3 µm (mean±SEM, n=23) and that in the 7 h group was 174.8±3.6 µm (n=8, p<0.001). The reduction in overall retinal thickness was mainly a result of a thinning of the inner retina layers (IPL to ILM, Figure 3A-C). The thickness of the inner retinal layer in the control group was 90.2±0.6 µm (n=23), and that in the 7 h group was 63.2±2.2 µm (n=8; p<0.001). Ocular hypertension for up to 7 h did not affect the thicknesses of the ONL, OPL, or INL (Figure 3A,D-F). Significant cell loss in the GCL was observed in all three experimental groups (5 h: 8.6±0.5 cells/300 µm; 6 h: 7.8±0.5 cells/300 µm; 7 h: 6.2±0.7 cells/300 µm; all p<0.001) compared to the control group (12.4±0.4 cells/300 µm; Figure 3A, G). These changes in the retina confirm the duration-dependent ON damages induced by elevated IOP.

### Loss in DTMR-Labeled RGCs Induced by IOP Elevation

**Figure 4:**
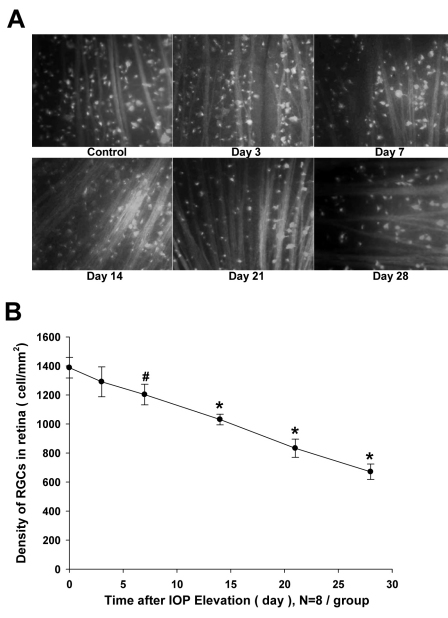
Effect of ocular hypertension (45 mmHg for 7 h) and RGC loss over 28 days. DTMR-labeled retinal ganglion cell (RGC) density was examined at designated time points after IOP elevation. **A**: Representative 40× images from the central retina, showing a time-dependent loss of RGC. **B**: Quantitative comparison of RGC densities between a naïve rat (Time 0) and different days after ocular hypertension (45 mmHg for 7 h). Symbols represent mean±SEM (n=6). ^#^: p<0.05 versus control using a one-way ANOVA and a Dunnett’s test. *: p<0.001 versus control using a one-way ANOVA and a Dunnett’s test.

To corroborate the ocular hypertension-induced loss of cells in the GCL, DTMR-labeled RGC counts were performed on retina flatmounts derived from eyes in which the IOP was elevated to 45 mmHg for 7 h. Figure 4A shows representative images of retinas at different time points, from 3 days to 28 days, after a 7 h, 45 mmHg IOP elevation. It is clear from these images that progressive RGC loss was obvious after the insult. Quantitative analysis of this information is presented in Figure 4B. Thus, the density of DTMR-labeled RGC in the control retinas was 1388±71/mm^2^. Three days after IOP elevation, its density decreased, though not to the statistically significant 1291±103/mm^2^ (n=8, p>0.05). The RGC densities continued to decline. On Day 7, RGC density was 1203±71/mm^2^ (p<0.05). On Day 14, it was 1031±37/mm^2^ (p<0.001). On Day 21, it was 833±63/mm^2^ (p<0.001). Finally, on Day 28, it was 671±53/mm^2^ (p<0.001). Compared to the control group, these changes correspond to a 7%, 13%, 27%, 40%, and 52% RGC loss on Days 3, 7, 14, 21, and 28, respectively.

### IOP Elevation and electroretinography

To evaluate if the IOP elevation of 45 mmHg for 7 h affected outer retina functions, ERG was performed on insulted animals on Days 2, 6, 13, 20, and 27. Table 1 shows the amplitudes of A- and B-waves were not significantly affected compared to their respective baseline values (p>0.05). These findings suggest the outer retina was not functionally damaged by this procedure, which confirms the morphological findings shown in Figure 3.

**Table 1:** Effect of IOP Elevation (45 mmHg for 7 h) on ERG A- and B-wave amplitudes (mean±SEM; n=6)

**Time points**	**A-wave amplitude (µV)**	**B-wave amplitude (µV)**
Baseline	453±19	1180±79
Day 2	467±21	1089±83
Day 6	471±11	1293±113
Day 13	415±39	1228±32
Day 20	419±79	1057±91
Day 27	398±63	1001±109

Note: Data are mean±SEM (n=6). No statistical significance among the groups was detected by One-Way ANOVA.

### Protection by JNK Inhibitor SP600125

**Figure 5:**
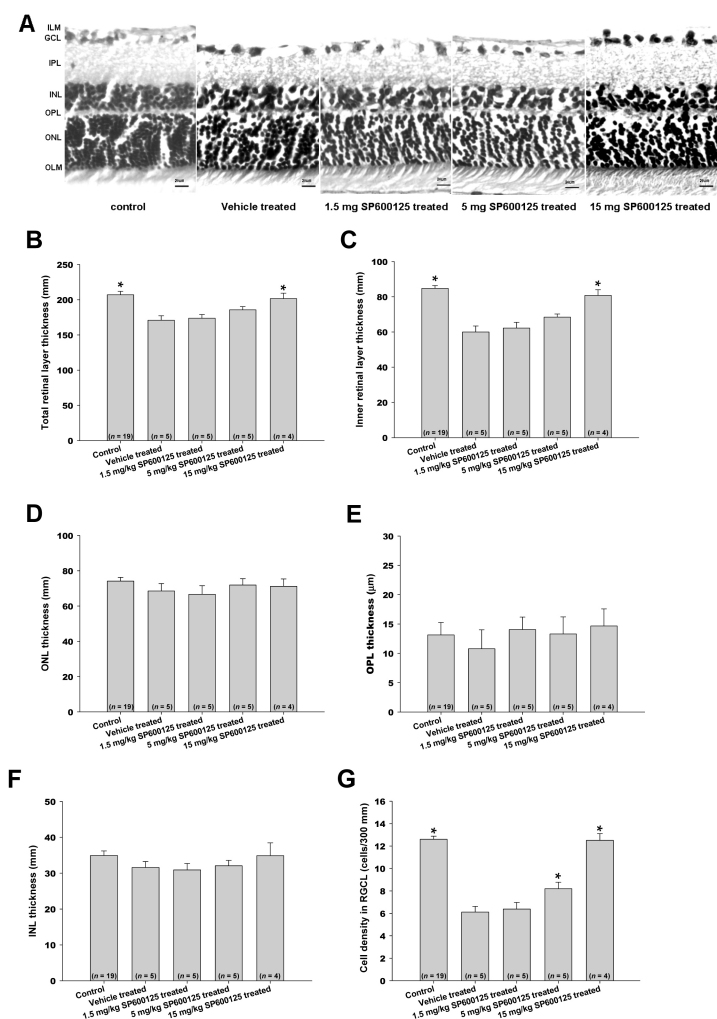
Dose-dependent protective effect of SP600125 against retina changes induced by intraocular pressure (IOP) elevation (45 mmHg) for 7 h. **A**: Representative images of hematoxylin and eosin-stained retinal cross-sections of eyes subjected to the different treatments. Thicknesses of the overall retina (**B**), inner retinal layer (**C**), ONL (**D**), OPL (**E**), INL (**F**), and cell density in the GCL (**G**) were quantified and are shown. Bars represent mean±SEM *: p<0.001 versus the “Control + Vehicle” group using a one-way ANOVA and a Dunnett’s test.

To investigate the potential neuroprotective effect of the JNK inhibitor against 45 mmHg ocular hypertension-induced injuries in the retina, a duration of 7 h was chosen because it produced the most severe damage of the conditions tested. In this study, three doses of SP600125 were tested (1.5, 5, and 15 mg/kg, i.p.). At the highest dose (15 mg/kg), SP600125 significantly reversed changes of retinal layer thickness produced by ocular hypertension. For example, the overall retinal thickness in the SP600125-treated ocular hypertensive eyes was 201.5±9.1 µm (n=4), which was significantly (p<0.001) thicker than that of the vehicle-treated ocular hypertensive eyes (170.9±8.2; n=5). However, it was not different from that of the naïve, ocular normotensive eyes (207.1±4.40; n=19; Figure 5A,B). The thickness of the inner retina in the SP600125-treated ocular hypertensive eyes was 80.8±3.7 µm (n=4), which was significantly (p<0.001) thicker than that of the vehicle-treated ocular hypertensive eyes (60.0±3.8, n=5). However, it was not different from that of the naïve, ocular normotensive eyes (84.8±1.36, n=19; Figure 5A,C). Similarly, cell density in the GCL also reflected the protective effect of the compound. The GCL cell density in the SP600125-treated ocular hypertensive eyes was 12.5±0.7 cells/300 µm (n=4), which was significantly higher (p<0.001) than that of the vehicle-treated ocular hypertensive eyes (6.1±0.8, n=5). However, it was not different from that of the naïve, ocular normotensive eyes (12.7±0.24, n=19; Figure 5A,G). At a lower concentration (5 mg/kg), SP600125 also significantly increased cell density in the GCL (p<0.001). At 1.5 mg/kg, the compound did not affect any of the parameters. Ocular hypertension, with or without treatment, did not significantly affect the thickness of the ONL, OPL, or INL (Figure 5A,D-F).

**Figure 6:**
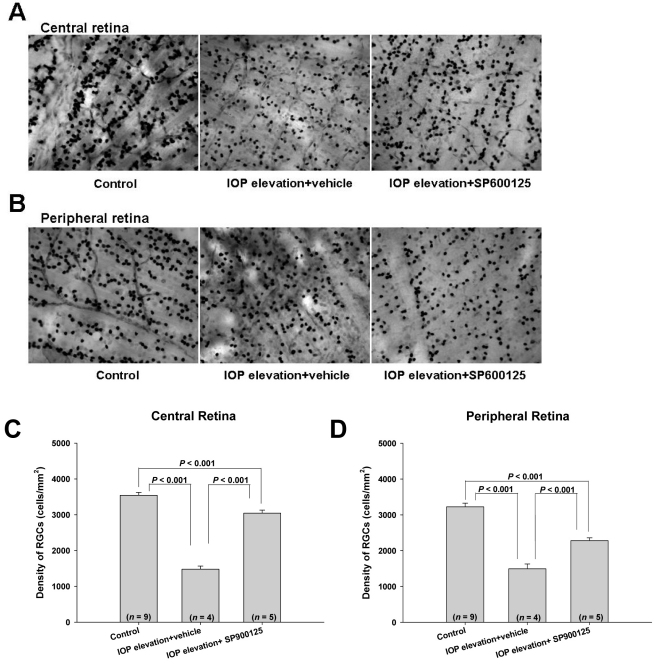
Protective effect of SP600125 on ocular hypertension-induced changes in retinal ganglion cell (RGC) density in the rat retina; “IOP elevation” corresponds to raising the intraocular pressure (IOP) to 45 mmHg for 7 h. **A**, **B**: Representative 40× images from the central (**A**) or peripheral (**B**) fields. The left-hand images are from control rats. The middle images are from IOP elevation + vehicle-treated rats. The right-hand images are from IOP elevation + SP600125 treated rats. Densities of RGC in the central (**C**) and peripheral (**D**) fields of the different study groups were quantified. Data are expressed as mean±SEM. Statistical significance was evaluated using a one-way ANOVA and a Bonferroni test.

To attempt to obtain a more accurate assessment of the effects of ocular hypertension with or without SP600125 on RGC survival, retina flatmounts from treated eyes were immunolabeled with antibody to Brn-3a, a specific marker for RGCs [21]. The labeled RGCs of one central and one peripheral field (40× magnification; area of each field=0.092 mm^2^) from each quadrant were counted manually. The counts from the four central fields of each retina were averaged and the mean RGC density (cells/mm^2^) was calculated and reported for each retina. Likewise, the counts from the four peripheral fields of each retina were assessed and reported in an identical fashion. Figure 6A,B show representative images of labeled RGCs in central and peripheral fields of control and ocular hypertensive rats treated with intraperitoneal administration of the vehicle or SP600125. Figure 6C,D summarize the quantification of RGC densities under various conditions. In the central retina of control eyes, there were 3542±85 RGCs/mm^2^ (n=9). Ocular hypertension for 7 h reduced RGC survival and significantly (p<0.001) lowered the RGC density to 1481±99 cells/mm^2^ (n=4), whereas treatment with SP600125 partially protected against this insult and significantly (p<0.001) increased the RGC density to 3044±97 cells/mm^2^ (n=5). Similar findings were observed for the peripheral retina. Ocular hypertension significantly diminished the RGC density to 1496±152 cells/mm^2^ (n=4), compared to that of the control retinas, which was 3225±108 cells/mm^2^ (n=9, p<0.001). SP600125 significantly (p<0.001) increased the RGC density to 2282±88 cells/mm^2^ (n=5).

## Discussion

In this report, we show that the suture-pulley model elevates IOP dependent on the standard weight (in grams) applied to the eye. Specifically, when the standard weight increases, IOP increases correspondingly. Prolonged elevation of IOP to 45 mmHg for 5–7 h induced irreversible damage to the RGC—as indicated by a significant loss of RGC, thinning of the inner retinal layer, and optic neuropathy—without affecting the outer retina. These effects are similar to those observed in acute angle-closure glaucoma attacks. We further demonstrated that systemic administration of the JNK inhibitor SP600125 significantly protected against ocular hypertensive-induced RGC loss.

As previously reported [13], the current suture-pulley method that gently compresses the eye to increase IOP is not invasive and is technically very easy to implement. It is not an excessively delicate procedure, so sophisticated and lengthy training is not required. Prior to the current study, we used this technique to induce transient retinal ischemia using a 35 g weight, as indicated by blanching of the retina during the procedure, and the diminished amplitudes of A- and B-waves [13]. Subsequently, we found that by decreasing the weight, we can reproducibly generate moderate elevation of IOP without affecting retinal blood flow. Therefore, this method is useful for studying acute ocular hypertension, such as acute PACG attacks. We targeted IOP at 45 mmHg to function as a glaucomatous insult to RGCs because various studies determined that 30–50 mmHg IOP is the threshold of selective damage to RGCs. This is further corroborated since an IOP of 50 mmHg has been observed to selectively impair optic nerve oxygenation without affecting choroidal supply (outer retina) [25]. However, most of these insults only produced a transient, reversible functional change of the inner retina or RGC, without affecting the long-term function or survival of RGCs. Our findings indicate that increasing the duration of 45 mmHg IOP to 5–7 h was sufficient to produce irreversible damage to ON axons and RGCs, without injuring the outer layers of the retina. The decrease in ON axons and RGC density correlated with the duration of hypertension, as indicated by the ONDS, GCL cell density, retinal layer thickness, and DTMR-labeled RGC density studies. Based on these results, we further selected a 7 h duration of hypertension as our standard study protocol because it caused the maximum damage within a practical time frame for an experimental procedure. The pressure-induced RGC damage was not immediately apparent after the insult; the loss of RGC as assessed by DTMR-labeled cells in the retina became more severe as the post-procedure time lengthened, such that approximately 50% of RGCs vanished 28 days later.

The prolonged application of moderate ocular hypertension allows investigation of the dynamics of initial morphological, molecular, and functional changes under controlled conditions, which provides insight into the effects of moderate short-term elevated IOP on RGCs and the possible underlying mechanisms of RGC damage during the early stages of glaucoma.

Many mechanisms could be responsible for RGC injury induced by elevated IOP. Apoptosis was observed in the GCL following IOP elevation [26]. The neurodegenerative effect demonstrated by this method was likely the result of apoptosis in RGCs [22]. At the present time, it is not clear where the initial primary injury site is. The excessive pressure may damage the RGC soma directly, but it can also initiate damage by compressing the RGC axons, which may interfere with intra-axonal transport of pro-survival molecules, such as trophic factors. Alternatively, pressure-induced compression of the retinal blood vessels can cause mild ischemia in certain retinal tissues [12-14]. For example, the inner retina, which has a high metabolic demand and the blood flow of which is supplied by the central retinal artery, may be more vulnerable to metabolic stress induced by the insult when compared to the outer retina [12-14].

There is a well recognized need to develop glaucoma therapies that target mechanisms other than IOP control. Protecting the retina from glaucoma injury is as important as controlling IOP. For example, JNK inhibitors such as SP600125 have been shown to reduce neuronal cell death in the brain as well as the retina. Such inhibitors protect against rat hippocampal CA1 cell loss caused by transient brain ischemia/reperfusion [19,20]. SP600125 also protects against excitotoxicity-induced apoptosis of RGCs [19]. In the present study, we found that SP600125 significantly preserved RGC density in rats compared to the vehicle-treated group after 7 h of IOP elevation. The results of this study suggest that SP600125 interferes with the JNK cascade of events responsible for RGC apoptosis and supports RGC survival.

In summary, the results of this study demonstrate that the progressive loss of RGC over the course of weeks and the decrease in inner retinal thickness are a direct response to the prolonged duration of applying 45 mmHg IOP to the rat eye. SP600125 protects RGCs from this insult, indicating that JNK activation is a key signaling component that contributes to RGC loss in this model and may be a potential neuroprotective target for the treatment of PACG attacks or other forms of glaucomatous optic neuropathy and retinopathy.
